# Sport Fields as Potential Catalysts for Physical Activity in the Neighbourhood

**DOI:** 10.3390/ijerph9010294

**Published:** 2012-01-19

**Authors:** Nicoleta Cutumisu, John C. Spence

**Affiliations:** 1 Sedentary Living Laboratory, Faculty of Physical Education and Recreation, University of Alberta, Van Vliet Centre, Edmonton, Alberta T6G 2H9, Canada; Email: jc.spence@ualberta.ca; 2 Alberta Centre for Active Living, 3rd Flr, 11759 Groat Road, Edmonton, Alberta T5M 3K6, Canada

**Keywords:** urban form, physical activity, sport fields, accessibility, Geographic Information Systems, enhanced 2 Step Floating Catchment Area (E2SFCA) method

## Abstract

Physical activity is associated with access to recreational facilities such as sports fields. Because it is not clear whether objectively- or subjectively-assessed access to facilities exerts a stronger influence on physical activity, we investigated the association between the objective and perceived accessibility of sport fields and the levels of self-reported physical activity among adults in Edmonton, Canada. A sample of 2879 respondents was surveyed regarding their socio-demographics, health status, self-efficacy, levels of physical activity, as well as their perceptions of built environment in relation to physical activity. Neighbourhood-level data were obtained for each respondent based on their residence. Accessibility to facilities was assessed using the enhanced Two-Step Floating Catchment Area method. Geographic Information Systems were employed. A logistic regression was performed to predict physical activity using individual- and neighbourhood-level variables. Women, older individuals, and individuals with higher educational attainment were less likely to be physically active. Also, individuals with higher self-efficacy and higher objectively-assessed access to facilities were more likely to be physically active. Interventions that integrate provision of relevant programs for various population groups and of improved recreational facilities may contribute to sport fields becoming catalysts for physical activity by generating movement both on the site and in the neighbourhood.

## 1. Introduction

The physical environment plays an essential role in shaping population health [1]. Neighbourhood environment and access to recreational facilities and public open spaces are associated with physical activity [2,3]. The active living research agenda has generated a multitude of studies focusing on the provision, access, use, and features of recreational facilities that not only encourage various structured and unstructured physical activities at the site, but also encourage neighbourhood walking [4,5]. For instance, neighbourhood environments that are perceived as providing good access to facilities for physical activity are conducive to individuals being active [6]. Research indicates that changes in the physical environment, such as renovating parks and enhancing access to multiuse trails can be effective in increasing physical activity in urban centres [7]. Given that a large majority of Canadians are not physically active enough to experience health benefits [8] and that the majority of Canadians live in urban areas [9], the role of built environment is an important public health issue. To help develop such interventions, it is necessary to elucidate whether accessibility of recreational facilities (defined as the ease of reaching desired activities; indicating both the distribution of activities offered by facilities and the travel to these activities; [10]) is associated with levels of physical activity in urban populations. 

Because it is not clear yet whether *objective* or *subjective* environments exert a stronger influence on physical activity, both objectively-assessed and subjectively-assessed accessibility need to be considered. Objectively-assessed accessibility is typically based on Geographic Information Systems (GIS) databases (e.g., using catchments around facilities and individuals’ homes). Subjectively-assessed accessibility is based on individuals’ perceptions in terms of availability of facilities (e.g., whether a person considers they have access to recreational facilities in their neighbourhood). Also, because current evidence shows that *extra-personal* influences, such as influence of access to facilities, on physical activity are mediated by individual-level factors, such as age, gender, SES and cognitive factors (e.g., perceptions, motivations, attitudes), both *extra-personal* and *intra-personal* influences need to be considered together in order to gain a better understanding of physical activity [11]. In particular, self-efficacy (defined as the sense of personal agency about one’s ability to perform a specific behaviour; [12]) is one cognitive factor that is most consistently positively associated with physical activity and that, therefore, merits consideration. 

The purpose of this study was to investigate the association between the objective and perceived accessibility of facilities for physical activity and the levels of physical activity among adults in Edmonton, Alberta, Canada. We hypothesized that accessibility of sport fields would be associated with individuals’ physical activity independent of individual-level psycho-social and socio-demographic correlates, such as age, gender, SES, and self-efficacy.

Accessibility has been a subject of interest for researchers investigating spatial distribution of basic services such as health services [13], food establishments [14,15], as well as recreational facilities [16,17]. Accessibility quantifies the spatial distribution of relevant opportunities available within a selected area and the spatial separation between an origin of interest and these opportunities [18,19]. Origins and destinations are operationalized as either home-based postal code centroids or spatial units, such as census tracts, each carrying a different set of consequences for the analysis [18,20]. Separation between origins and destinations is operationalized most often using Euclidean distance (straight-line) and street network distance (along routes). Typically, the calculation of accessibility is restricted to an area of interest only (by selecting a certain threshold distance to define that specific area of interest), so only a selected number of facilities are considered, as well as of the cost of travel to these selected facilities. Although it is clear that distance to facilities seems to influence associations between availability of facilities and levels of physical activity, with better access to attractive facilities being associated with more physical activity [21], it is not clear what threshold distance is optimal to employ in defining the area of interest to calculate accessibility of facilities for physical activity. As noted by Kaczynski and Henderson [22], most studies addressing the availability of facilities to adults have chosen a search radius varying between 400 m to 1500 m. It appears that associations at the local level (400 m–800 m) are stronger for public open spaces and playgrounds, while associations at the regional level (beyond 800 m) are stronger for formal facilities for physical activity (such as fitness, sport, and recreational facilities) or for natural environment (such as beaches, rivers, and open spaces)—see [21,23]. Diez-Roux and her colleagues [23] suggest 1 mile (1600 m) as an appropriate distance for studying the availability of recreational facilities.

Even though most studies report positive associations between access to various types of settings (such as parks, open spaces, and community recreational facilities) and physical activity levels in adults (see review by Kaczynski and Henderson [22]), some studies report inconsistent associations or no association for access to recreation centres [24,25]. Generally, open areas show stronger associations with physical activity than built facilities, although findings are still inconclusive [22]. It is likely that measurement of spatial accessibility to facilities is an explanation for these inconclusive findings. Therefore, more studies are necessary that employ better measures of spatial accessibility, in particular objective measures of assessing accessibility, to better understand physical activity patterns in urban populations.

One of the most frequently-used operationalizations of objectively-assessed accessibility is based on the gravity potential model [19], which involves an assessment of the availability of a set of destinations weighted by impedance (a function of travel cost from an origin to all destinations considered; also called a distance decay function). For our study we used the enhanced two-step floating catchment area method (E2SFCA; [26]), which is easy to apply and differentiates between areas where similar numbers of facilities serve zones with different population densities, thus providing a more realistic image of accessibility to facilities. 

## 2. Methods

### 2.1. Study Area

This study focused on residential neighbourhoods located in the city of Edmonton, the capital of the Province of Alberta, Canada. Data for SES and walkability were available for only 195 residential neighbourhoods (M = 1.09 km^2^, SD = 0.38). City of Edmonton provided data on neighbourhood profiles, which were compiled by Edmonton Social Planning Council using information from the 2001 Census. Also, data on the centroids of the 2001 census blocks was obtained from Statistics Canada, along with population counts for census blocks, which were extracted from the GeoSuite 2001 software by Statistics Canada. Census blocks are small geographic areas delimited by roads or census geographical areas. In addition, the 2001 Postal Code Conversion Files (PCCF) produced in 2003 [27] by Statistics Canada was used because it enabled the spatial representation of Edmonton’s postal codes. Data on neighbourhood crime and traffic incidents for the year of 2002 were obtained from the Edmonton Police Service report [28]. Road network data were provided by the CanMap^®^ Route Logistics v.8.2. (2004) package produced by DMTI (Desktop Mapping Technologies Inc.). Data on neighbourhood boundaries and the locations of the centroids of 362 sport field complexes were obtained in 2005 from GeoEdmonton. These complexes are defined as facilities for physical activity that include outdoor sport fields, such as diamonds, rectangular fields, and tracks. Some additional indoor facilities, such as school gyms, hockey rinks, and swimming pools, may be present, but most of the activities take place outdoors. 

### 2.2. Participants

The original sampling frame for this study consisted of 4175 adults living in the Capital Health region of Edmonton, Alberta, who took part in the Population Health Survey 2002 (PHS). But, only those individuals living in the 195 residential neighbourhoods within the region (*n* = 2879) were included in this study. Table 1 indicates the median age for our respondents is within the 35–49 years old group and the median family income for our respondents is within the $40,000–59,999 group. This is consistent with the 2001 Census of Canada, which reported the median age for Edmontonians was 35.3 (34.4 for males and 36.2 for females) and the median family income was $56,212. Respondents were reached through random digit dialing between October 28th and December 15th, 2002. The survey provided data on leisure-time physical activity levels, perceived environment, and socio-demographics. The location of the respondents’ households was determined based on the postal code centroids for the addresses of the respondents, using the Postal Code Conversion Files (PCCF) produced by Statistics Canada [27]. It was assumed that each respondent’s household was located in the postal code centroid corresponding to each household. 

### 2.3. Measures

To capture the association between objective and subjective environments and physical activity, *individual* and *neighbourhood-level measures* were employed. Individual-level measures included individual socio-demographics, self-reported physical activity, self-efficacy, objective accessibility to sport field complexes, and perceptions of the neighbourhood environment (such as perceived access to facilities for physical activity, perceived risk from crime, and perceived risk from traffic). Neighbourhood-level measures included neighbourhood actual risk (from crime and from traffic) and neighbourhood SES.

#### 2.3.1. Individual Socio-Demographics

Participants in the 2002 PHS survey were asked to report their age (in years), gender, whether or not they had a health issue that would prevent them from taking part in physical activity, and whether any children under 18 lived in the household. Individual-level education and income were also recorded. 

#### 2.3.2. Self-Reported Physical Activity

PHS 2002 employed the short form of the International Physical Activity Questionnaire (IPAQ) to measure physical activity in various domains (e.g., leisure-time, domestic, and gardening activities). Good reliability (*r* = 0.83) and validity data correlated with accelerometer measurements (*r* = 0.52) are available for this scale [29]. Questions about walking, moderate-intensity, and vigorous-intensity activities performed for at least 10 min during the week prior to the survey were the basis for calculating separate weekly duration scores for each type of activity. By weighting each type of activity according to its corresponding MET (multiples of the resting metabolic rate), a score in MET*min was calculated. The following average MET*min values per week were created: (1) the average MET*minu/week for walking was calculated as 3.3 × walking min × walking days per week; (2) the average MET-min/week for moderate-intensity physical activity was calculated as 4.0 × moderate-intensity activity min × moderate-intensity days per week; and (3) the the average MET-min/week for vigorous-intensity physical activity was calculated as 8.0 × vigorous-intensity activity min × vigorous-intensity days per week. A score for total physical activity accumulated per week was calculated as the sum of MET*min per week scores for walking, moderate-intensity, and vigorous-intensity physical activity. Based on the recommendations of Haskell and colleagues [30], respondents were assigned to one of two categories: *insufficient physical activity* or *sufficient physical activity*. Specifically, a cut-off value of 750 MET*min per week was used to determine level of activity. This reflects the recommendations that health benefits can be achieved by accumulating a minimum of between 450 and 750 MET*mins per week of combined moderate and vigorous physical activity [30]. 

#### 2.3.3. Self-Efficacy

Self-efficacy was assessed using a 100-point scale (divided into increments of 10 units), in which 0 means *Completely certain I cannot do it* (participate in regular physical activity) and 100 means *Completely certain I can do it* (participate in regular physical activity). The validity [31] and reliability [32] of this scale for exercise have been extensively documented.

#### 2.3.4. Objective Accessibility of Sport Field Complexes

The E2SFCA was employed to assess objectively accessibility to complexes of sport fields. A threshold of 1500 m was chosen for the accessibility calculations to reflect pedestrian distance based on the rationale that it represents the equivalent of a 15-min pedestrian trip. Restricting our analysis to 1500 m should be appropriate, because distances between 800 m and 2000 m have been used to capture the relationship between access to facilities and physical activity for individuals who tend to rely more on local facilities, as well as for individuals who are willing to travel to multiple places located outside of their local environments [21,23,33].

The E2SFCA method involved calculations based on two floating catchments, which were calculated in two ways: (1) Euclidean (straight-line) distance, and (2) based on street network distance. In the first step, the *complex catchment* (*i.e.*, the catchment area of a sport complex) was created for all sport complexes using the centroid of each complex. In the case of street network distance calculations, the distance along the network was considered using as origin the point on the network located nearest to the facility’s centroid and as destination the point on the network located nearest to the census block’s centroid. Three travel zones, denoted *D*_1_ (0–500 m), *D*_2_ (500–1000 m), and *D*_3_ (1000–1500 m), were defined within each catchment based on three distance increments of 500 m created around the location *j* of a sport complex. Different weights (*W*_1_, *W*_2_, and W_3_, respectively) were assigned to the population present in each of the three travel zones within each catchment. These weights were used in the computation of the *complex-to-population* ratio at the location *j* of a sport complex, based on the following formula:


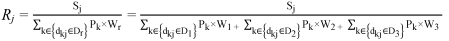


where *S_j_* represents the *supply of sports complexes*, *P_k_* represents the population at census block *k* located within each of the three travel zones *D_r_* (*r* 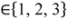), *d_kj_* represents the distance between the centroid of the census block *k* and the centroid of the sports complex *j* (which was measured using Euclidean distance and street network distance, respectively), and *W_r_* (*r* 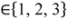) represents the weight associated with each travel zone. We considered *S_j_* to be 1, which represents the number of sports complexes at that location. The value of *R_j_* quantifies the ratio of *supply* (sport complexes) to *demand* (census block population), while restricting the number of complexes and census blocks that were taken into account. The *W_r_* weights were calculated using the impedance function *W_r_ = f(d_r_)*, where *d_r_* represents the distance from *j* to the *r*-th travel zone. 

Typically, the weights associated with each travel zone are calibrated using actual survey data, which were not available in the case of the use of sports complexes and in the case of travel to sport complexes. For such situations, an impedance function can be used to express the cost of travel [34]. Luo and Qi [26] employed a Gaussian function as a specification of the impedance function in a study based on motorized travel between zones. Iacono, Krizek, and El-Geneidy [35] suggest that a negative exponential specification of the impedance function may be appropriate to use in the context of non-motorized travel because it provides a slower decay rate and it better accounts for shorter distances such as walking distances. Cohen *et al.* [36] employed both a Gaussian and a negative exponential function for the specification of the impedance functions to derive the β coefficients necessary to capture the distance decay rate in modeling the access of girls to parks located within a mile from the girls’ homes, by solving for β coefficients that correspond to a value of 0.5 for each of the functions used. Thus, Cohen *et al.* assigned a weight of 0.5 to a park that was located at a distance of 1 mile from the girls’ homes. 

We employed a negative exponential function as a specification for the impedance function, to derive the β coefficient that corresponds to a value of 0.1 for the negative exponential function at a 1.5 km distance. We chose this function value of 0.1 at 1.5 km to determine the set of *W_r_* weights because recent studies have used similar impedances in the case of accessibility calculated for non-motorized travel and calibrated based on local travel data [35,37]. Also, in the case of Edmonton, the 2005 Edmonton Household Travel survey [38] indicates that the proportion of walk trips is 11% and the average walking trip length is 1 km. In addition, a recent study in Montreal [37] found the 85% percentile of pedestrian travel for leisure purpose was 1403 m. Therefore, we assumed Edmontonians’ average walking length to a leisure destination would be longer, as well, and we chose a value of the negative exponential function of 0.1 at a distance 1.5 km. Furthermore, we chose the β coefficient so that the value of the function decreases in half as the distance doubles. For example, the probability that a person would travel to a sports complex located within 500 m away from home would be 1. This probability decreases by almost a half for sports complexes located between 500 and 1000 m to a value of 0.46. Further, probability decreases by almost a half for sports complexes located between 1000 and 1500 m to a value of 0.22. Thus, we performed the E2SFCA calculations using a β coefficient of 1.53 corresponding to a value of the negative exponential function of 0.1 at a distance of 1.5 km based on Euclidean, as well as on street network distance. This corresponds to the following set of *W_r_* weights: *W*_1_ = 1 (for travel zone *D*_1_), *W*_2_ = 0.46 (for travel zone *D*_2_), and *W*_3_ = 0.22 (for travel zone *D*_3_). 

In the second step, the *population catchment* (the area surrounding a population location; e.g., the census block centroid) was created, consisting of three catchments using the same 500, 1000, 1500 m increments that were used in the first step. The E2SFCA accessibility was calculated as the *population-to-complex* value that represents the sum of the *complex-to-population* ratios (calculated in the first step) for all complexes located within the population catchment. The value for the E2SFCA accessibility 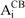 of the census block at the location *i* was calculated using the formula:


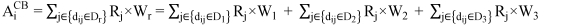


where *R_j_* represents the *complex-to-population* ratio at complex *j* located within the catchment around census block *i*, that is 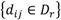, *dij* represents the distance between *i* and *j,* and the *W_r_* (*r* 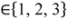) represents the same weight associated with each travel zone as the weight used in the computations performed in the first step.

Finally, the accessibility of the postal code of the respondents at location *p* was calculating by assigning the E2SFCA accessibility value corresponding to the census block located nearest to the centroid of the respondent’s postal code.

#### 2.3.5. Perceptions of Neighbourhood Environment

The PHS survey included the International Physical Activity Prevalence Study’s Environmental Survey Module (IPS), which was used in conjunction with IPAQ. Respondents’ neighbourhoods were defined as areas around their homes to which they could walk in 10–15 min. The IPS survey included 17 items regarding the type of housing, access to neighbourhood facilities, access to public transit stops, presence of pedestrian infrastructure, access to free or low-cost recreational facilities, safety from crime and traffic, presence of active people in the neighbourhood, neighbourhood aesthetics, intersection density, and car ownership. Most questions from the IPS were adapted from previously-used instruments that demonstrated good validity and reliability [39,40]. 

Only variables measuring access to free or low-cost recreational facilities (*perceived access*) and safety from crime (*perceived risk from crime*) and from traffic (*perceived risk from traffic*) were employed. The questions regarding these items were rated using a four-point Likert response scale ranging from *strongly disagree* to *strongly agree*. In addition, a *don’t know/not sure* option was included for all variables. *Perceived access* (one item) responses ranged from *strongly disagree* to *strongly agree* with the statement *My neighbourhood has several free or low cost recreational facilities. Perceived risk* variables ranged from *strongly disagree* to *strongly agree* with the statements *The crime rate in my neighbourhood makes it unsafe to go for walk at night* (*perceived risk from crime;* one item) and *There is so much traffic on the streets making it difficult or unpleasant to walk in my neighbourhood* (*perceived risk from traffic*; one item). The *strongly disagree* and *disagree* categories were collapsed into a single category of *disagree.* Also, the *strongly agree* and *agree* categories were collapsed into a single category of *agree.*

#### 2.3.6. Neighbourhood Actual Crime Levels (Risk)

Two variables were included to represent neighbourhood actual levels of risk from crime and traffic. Risk from crime was assessed based on the number of incidents in the following categories: violent crime (e.g., assaults and robbery) and property crime (e.g., break and entry, motor vehicle, and other theft), expressed as the percentage of violent and property crime incidents per neighbourhood population. Risk from traffic was based on the number of criminal code traffic violations (e.g., dangerous driving, failure to remain at the accident scene) expressed as the percentage of traffic violations incidents per neighbourhood population. 

#### 2.3.7. Neighbourhood Socio-Economic Status (SES)

Neighbourhood-level SES was based on data extracted from the 2001 Canadian Census and compiled by the Edmonton Social Planning Council. Neighbourhood SES was calculated as a sum of z-scores of net educational level and median income of census families ($) minus the z score of the proportion of unemployed using a procedure by Demissie, Hanley, Menzies, Joseph, and Ernst [41]. 

### 2.4. Analysis

We used ArcGIS 9.3.1 (ESRI, Redlands, CA) and its Network Analyst extension to assess the E2SFCA accessibility to sport field complexes using the Route Logistics file by DMTI. SPSS 17 (version 17, Chicago, IL) was employed for the statistical analyses. The level of statistical significance was set at *p* < 0.05. A hierarchical logistic regression was performed, in which self-reported levels of physical activity (*sufficiently active* vs. *insufficiently active*) were regressed on individual socio-demographic variables, actual and perceived access to sport fields complexes, actual and perceived crime rates, neighbourhood SES, and self-reported self-efficacy. Three models were created to examine the role of the selected predictors in influencing self-reported physical activity. In Model 1, we included only individual socio-demographic characteristics. In Model 2, we added perceived environment variables. Finally, in Model 3, we added objective environment variables. 

## 3. Results and Discussion

Descriptive statistics are provided in Table 1. As illustrated in Table 2, approximately 23% of respondents were classified as reporting insufficient physical activity (sedentary and light activity) and 77% were classified as reporting sufficient physical activity (moderate and heavy activity). 

**Table 1:** Sample characteristics.

Sample characteristics		Frequency	Valid Percent
Gender			
	Male	1415	49.1
	Female	1464	50.9
		*n* = 2879	100.0
Age (years)			
	18–24	610	21.2
	25–34	605	21.0
	35–49	556	19.3
	50–64	553	19.2
	65+	555	19.3
		*n* = 2879	100.0
Education			
	Less than high school	405	14.1
	Completed high school	599	20.9
	Incomplete post-secondary	523	18.2
	Completed non university	597	20.8
	Completed university	533	18.6
	Post-Bachelor university	213	7.4
		*n* = 2870	100.0
Income			
	<$20,000	440	19.1
	$20–39,999	626	27.2
	$40–59,999	484	21.1
	$60–79,999	312	13.6
	$80–99,999	163	7.1
	$100,000+	273	11.9
		*n* = 2298	100.0
Health condition			
	Yes	528	18.4
	No or Not Applicable	2339	81.6
		*n* = 2867	100.0
Children under 18 at home			
	Yes	865	30.1
	No	2011	69.9
		*n* = 2876	100.0
Neighbourhood has access to free/low cost facilities			
	Disagree	489	17.4
	Neither	363	12.9
	Agree	1961	69.7
		*n =* 2813	100.0
Crime rate makes neighbourhood unsafe for walking at night			
	Disagree	1499	54.4
	Neither	439	15.9
	Agree	819	29.7
		*n =* 2757	100.0
Traffic makes neighbourhood difficult/unpleasant for walking			
	Disagree	1967	68.8
	Neither	364	12.7
	Agree	527	18.4
		*n* = 2858	100.0

*Note:* Some variables have missing cases.

**Table 2:** Physical Activity.

Physical Activity	Frequency	Percent	Valid Percent	Cumulative Percent
Insufficient physical activity (< 750 MET*min per week )	663	23.0	23.0	23.0
Sufficient physical activity (750 MET*min or more per week)	2215	76.9	77.0	100.0
	*n* = 2878	100.0	100.0	

Table 3 presents the associations between self-reported levels of physical activity and individual-, as well as environmental-level variables corresponding to the separate analyses conducted using accessibility calculated based on Euclidean and on street network distance. Because accessibility based on Euclidean distance emerged as a significant predictor, unlike accessibility based on street network distance, we discussed only the results for analyses involving accessibility calculations based on Euclidean distance.

In Model 1, logistic regression indicated that women (OR = 0.61, 95% CI = 0.48, 0.77) were significantly less likely to be sufficiently physically active as recommended in comparison to men, which is consistent with previous research [42]. Consequently, interventions should focus on encouraging physical activity in women at various life stages (e.g., change in physical status, such as becoming a mother), particularly in younger women, to create habits for life.

Also, individuals aged 25–34 (OR = 0.57, 95% CI = 0.37, 0.85), 35–49 (OR = 0.62, 95% CI = 0.40, 0.95), 50–64 (OR = 0.57, 95% CI = 0.37, 0.88), and 65 and over (OR = 0.55, 95% CI = 0.35, 0.86) were less likely to be physically active as recommended, compared to younger individuals. This is consistent with previous work [42,43] and adds to the evidence that young adulthood is a critical time in declining physical activity levels and, therefore, it needs to constitute an impetus for public health interventions [44]. Interventions should encourage walking to recreational facilities that can be combined with exercise programs offered by the recreational facilities to improve physical activity in older age groups.

Unlike income, which did not emerge as a significant predictor in this study, education was associated with being sufficiently active as recommended. Compared with the group with the least education, individuals who did not complete post-secondary education (OR = 0.57, 95% CI = 0.36, 0.91), individuals who did complete non-university education (OR = 0.60, 95% CI = 0.39, 0.93), and individuals with post-bachelor level education (OR = 0.37, 95% CI = 0.22, 0.63) were less likely to be sufficiently physically active as recommended. This conflicts with previous findings [42] and may be due to the fact that total physical activity does not differentiate among various domains of physical activity. It is also possible that individuals with lower education are more likely to engage in highly active occupations that provide incentives to participate in various active after-work recreation (e.g., team sports), so these individuals accumulate more leisure-time physical activity than their counterparts with sedentary occupations, suggesting that interventions should target sedentary occupations [45]. 

In addition, having children under 18 living at home and having a health condition that prevents individuals from engaging in physical activity were not significantly associated with being sufficiently active. However, individuals having children under 18 living at home were more likely to be sufficiently active than their counterparts. Individuals having a constraining health condition were less likely to be sufficiently active than their counterparts. 

Respondents with higher levels of self-efficacy were 1.02 times (95% CI =1.02, 1.03) significantly more likely to be sufficiently active as recommended. These results are consistent with previous research [46]. Self-efficacy is thought to negotiate the constraints associated with physical activity (e.g., lack of opportunities and access to facilities; [47]). For instance, an environment with higher access to facilities may help increase self-efficacy in individuals, which would consequently increase physical activity levels [48,49].

None of the perceived environmental variables included in Model 2 were statistically significant. We found no association between perceived access to facilities and physical activity, contrary to studies that found perceived access to sport/fitness facilities predicting physical activity [50,51]. However, compared with individuals who perceived their environment as providing less access to free or low cost facilities, individuals who perceived their environments as providing more access to facilities were more likely to be sufficiently physically active. 

Also, perceived risk from crime and from traffic were not significantly associated with being sufficiently active as recommended. However, individuals who disagreed that crime was a problem were more likely to be sufficiently active than their counterparts. This is consistent with previous research [52,53]. 

**Table 3:** Contribution of predictors to explaining self-reported levels of physical activity.

Predictors	Analysis: Euclidean Distance—Negative Exponential Function						Analysis: Street Network Distance-Negative Exponential Function					
	Model 1		Model 2		Model 3		Model 1		Model 2		Model 3	
	OR	95% CI	OR	95% CI	OR	95% CI	OR	95% CI	OR	95% CI	OR	95% CI
**Gender**												
Men												
Women	0.61	(0.48, 0.77)	0.58	(0.46, 0.75)	0.58	(0.45, 0.74)	0.61	(0.48, 0.77)	0.58	(0.46, 0.74)	0.58	(0.46, 0.75)
**Age**												
18–24												
25–34	0.57	(0.37, 0.85)	0.55	(0.37, 0.84)	0.55	(0.36, 0.82)	0.57	(0.37, 0.85)	0.55	(0.37, 0.84)	0.55	(0.36, 0.83)
35–49	0.62	(0.40, 0.95)	0.59	(0.38, 0.91)	0.58	(0.38, 0.90)	0.62	(0.40, 0.95)	0.59	(0.38, 0.91)	0.59	(0.38, 0.91)
50–64	0.57	(0.37, 0.88)	0.56	(0.36, 0.87)	0.56	(0.36, 0.87)	0.57	(0.37, 0.88)	0.56	(0.36, 0.87)	0.57	(0.37, 0.89)
65+	0.55	(0.35, 0.86)	0.53	(0.34, 0.84)	0.51	(0.32, 0.81)	0.55	(0.35, 0.86)	0.53	(0.39, 0.84)	0.53	(0.33, 0.84)
**Education**												
Less than HS												
Completed HS	0.83	(0.54, 1.29)	0.85	(0.55, 1.32)	0.84	(0.54, 1.30)	0.83	(0.54, 1.29)	0.85	(0.55, 1.32)	0.85	(0.55, 1.32)
Incomplete post-secondary	0.57	(0.36, 0.91)	0.58	(0.36, 0.92)	0.58	(0.37, 0.93)	0.57	(0.36, 0.91)	0.59	(0.36, 0.92)	0.58	(0.37, 0.93)
Completed non-university	0.60	(0.39, 0.93)	0.61	(0.40, 0.94)	0.61	(0.40, 0.95)	0.60	(0.39, 0.93)	0.61	(0.39, 0.94)	0.61	(0.40, 0.95)
Completed university	0.71	(0.45, 1.13)	0.73	(0.46, 1.15)	0.71	(0.45, 1.13)	0.71	(0.45, 1.13)	0.73	(0.46, 1.15)	0.72	(0.45, 1.14)
Post-Bachelor university	0.37	(0.22, 0.63)	0.38	(0.22, 0.66)	0.37	(0.21, 0.64)	0.37	(0.22, 0.63)	0.38	(0.22, 0.66)	0.37	(0.22, 0.65)
**Income**												
<$20,000												
$20–39,999	0.83	(0.58, 1.18)	0.84	(0.58, 1.20)	0.85	(0.60, 1.22)	0.83	(0.58, 1.18)	0.84	(0.58, 1.20)	0.85	(0.59, 1.22)
$40–59,999	0.92	(0.62, 1.38)	0.96	(0.65, 1.44)	0.99	(0.66, 1.48)	0.92	(0.62, 1.38)	0.96	(0.65, 1.44)	0.99	(0.67, 1.50)
$60–79,999	0.81	(0.52, 1.27)	0.84	(0.53, 1.32)	0.86	(0.55, 1.36)	0.81	(0.52, 1.27)	0.84	(0.53, 1.32)	0.86	(0.54, 1.35)
$80–99,999	0.83	(0.49, 1.42)	0.86	(0.50, 1.47)	0.87	(0.51, 1.51)	0.83	(0.49, 1.42)	0.86	(0.50, 1.47)	0.88	(0.51, 1.52)
$100,000+	0.93	(0.57, 1.52)	0.95	(0.58, 1.57)	0.96	(0.58, 1.60)	0.93	(0.57, 1.52)	0.95	(0.58, 1.57)	0.96	(0.58, 1.59)
**Children under 18**												
Yes	1.31	(0.98, 1.75)	1.30	(0.97, 1.74)	1.32	(0.99, 1.77)	1.31	(0.98, 1.75)	1.30	(0.97, 1.74)	1.33	(0.99, 1.78)
No												
**Health condition**												
Yes	0.84	(0.61, 1.14)	0.82	(0.60, 1.12)	0.81	(0.59, 1.11)	0.84	(0.61, 1.14)	0.82	(0.60, 1.12)	0.81	(0.59, 1.11)
No												
**Self-efficacy**	1.02	(1.02, 1.03)	1.02	(1.02, 1.03)	1.02	(1.02, 1.03)	1.02	(1.02, 1.03)	1.02	(1.02, 1.03)	1.02	(1.02, 1.03)
**Perceived access**												
Disagree												
Neither			0.75	(0.49, 1.14)	0.73	(0.48, 1.11)			0.75	(0.49, 1.14)	0.74	(0.48, 1.19)
Agree			1.07	(0.78, 1.47)	1.04	(0.75, 1.43)			1.07	(0.78, 1.47)	1.03	(0.75, 1.43)
**Perceived risk from crime**												
Disagree			1.37	(0.93, 2.02)	1.41	(0.96, 2.09)			1.37	(0.93, 2.02)	1.43	(0.97, 2.11)
Neither			0.93	(0.70, 1.23)	0.92	(0.69, 1.23)			0.93	(0.70, 1.23)	0.93	(0.70, 1.25)
Agree												
**Perceived risk from traffic**												
Disagree			0.77	(0.50, 1.19)	0.77	(0.50, 1.19)			0.77	(0.50, 1.19)	0.79	(0.51, 1.21)
Neither			0.80	(0.58, 1.10)	0.82	(0.59, 1.13)			0.80	(0.58, 1.10)	0.83	(0.60, 1.15)
Agree												
**NSES**					1.08	(0.99, 1.17)					1.07	(0.99, 1.16)
**NRC**					0.98	(0.96, 1.01)					0.98	(0.96, 1.01)
**NRT**					**1.48**	**(1.01, 2.18)**					1.51	(1.03, 2.22)
**Accessibility**					**1.65**	**(1.01, 2.69)**					1.29	(0.94, 1.78)

*Note*: *p* < 0.05; HS = high school; NSES = Neighbourhood SES; NRC = Neighbourhood risk from crime; NRT = Neighbourhood risk from traffic.

In contrast, individuals who disagreed that traffic was a problem were less likely to be sufficiently active than their counterparts. One explanation for this finding is the fact that these individuals may live in areas with less traffic which are usually more characteristic of suburban environments, which may offer fewer opportunities for physical activity. It is also possible, as it was suggested in previous study [54], that individuals who are more active may have more opportunities to learn about their environments and thus, have more opportunities to notice issues related to the risk from traffic. 

Perceived environment variables did not emerge as predictors of physical activity in our study, perhaps because individuals tend to distort distances, likely from an evolutionary drive to survive by optimizing behaviour to conserve energy expenditure [55,56]. Therefore, if facilities are considered places that are desirable, distances to reach facilities from home are likely to be underestimated; if facilities are considered places that require higher energy expenditure, distances to reach facilities from home are likely to be overestimated. For instance, one study found that 37.6% and 29.4% of the respondents accurately estimated the distance of 1500 m to a sport field and a park, respectively; also, perceived distances to sport fields and parks were significantly overestimated [57]. 

In Model 3, individuals residing in neighbourhoods with higher risk from traffic were 1.48 times more likely (95% CI = 1.01, 2.18) to report sufficient physical activity as recommended. This may be explained by the fact that the areas with higher actual risk from traffic are located in the inner city, which is likely more conducive to physical activity than suburban areas. The fact that actual risk from traffic, unlike perceived risk from traffic, was significantly associated with physical activity could be attributed to measurement. Our measures of actual crime and traffic violation rates were aggregates calculated at the level of neighbourhood as an administrative unit, while our measures of perceived risk were assessed for the neighbourhood conceptualized as a buffer constructed around individuals’ postal codes. It is possible that our respondents may recall characteristics of areas that are smaller or larger than the buffers under discussion. In addition, it may be that individuals who perceive their neighbourhood as unsafe choose to use facilities in other areas [58]. In addition, although not significant, increased actual risk from crime was associated with a lower likelihood to be active as recommended, which is consistent with previous studies [53]. Similarly, neighbourhood SES was not associated with physical activity, although the direction of the association is consistent with previous research, with individuals living in more affluent areas being more likely to be active as recommended [52,59]. 

Individuals having higher access to sport fields were 1.65 times significantly more likely (95% CI = 1.01, 2.70) to report sufficient physical activity as recommended. This is consistent with previous research documenting the association between proximity to recreational facilities and physical activity in adults [48] and in youth [60,61]. The fact that objective, but not perceived environment, was associated with physical activity is consistent with recent research [62] and it suggests that individuals’ level of awareness about their environments may have played a role. This prompts a need to create awareness campaigns, especially amongst subgroups that are more likely to misestimate perceptions [63]. It also indicates that objective and subjective environments may influence behaviour independently [57,62]. Alternatively, methodological aspects of measuring environments are likely to have played a role in our findings, perhaps because of the conceptualizations of place that were used [33,64]. More exploratory studies are necessary to find better measures to capture place and to determine the size of the area in which environmental characteristics seem to exert the most influence on physical activity. 

Our study supports the definition of such an area using a street network distance radius of 1500 m. Although the associations with physical activity are small, our objective measure of access showed significance. Consistent with previous work in Edmonton [65], our study confirms that the provision of recreational facilities within a larger area (e.g., radii larger than 800 m) is associated with physical activity, and it also suggests that, while proximity matters for some activities that are more locally-bound, for other activities people are willing to travel further than their immediate proximity [33]. However, more exploratory studies are necessary to determine the extent to which distance influences the use of various types of facilities [66], in particular different types of recreational facilities.

Public health research documents that urban design land use policies, as well as creating or enhancing access to places for physical activity, may result in improvements in physical activity [7,67]. Thus, interventions are recommended to focus on creating more supportive environments for physical activity, which may be achieved in some cases by facility and site renovations [68]. Renovating under-utilized venues for outdoor physical activity could be a part of an urban renewal strategy of retrofitting aging structures (e.g., big-box malls, recreation centres, parking lots) throughout urban areas [69,70]. This is because such interventions have beneficial consequences for health and urban life in general [23]. By creating and fostering a sense of community [70], which is associated with improvements in physical and mental health [71], sport complexes can provide an avenue for active living for individuals of all ages. However, such interventions need to be complemented by a supportive system of streetscapes because it appears that a synergetic effect may occur between the presence of good quality recreational facilities and a safe, walkable, neighbourhood environment [72,73]. Just as commercial facilities enhance pedestrian movement [74], sport fields may exert a multiplicative effect as magnets for physical activity in the neighbourhood. Thus, future research should consider sport fields in conjunction with their surrounding environments [75,76]. 

This study has several strengths that are worth mentioning. It involved a representative sample of the population residing in Edmonton, Alberta, providing information that was assessed by employing validated measures of physical activity and perceived environment. It also involved an analysis that considered both individual- and neighbourhood-level variables and their association with physical activity. As well, this study used a composite measure of neighbourhood SES, because neighbourhood income or education alone may not be adequate proxies of SES in the context of Edmonton.

Some limitations may affect the results of this study. First, our outcome measure is a total physical activity measure that does not specifically address various domains of physical activity (such as recreation, work-related, household-related). We only included formal facilities for physical activity that are administered by the City of Edmonton because we did not have an exhaustive list of recreational facilities (e.g., private facilities) and trails. Also, because of the limited information available about the sports fields, we did not incorporate a measure of attractiveness in our assessments of accessibility. Thus, we assumed that all facilities are equally attractive [52] and well maintained and we did not consider the number of activities offered at the facility site, which seems to be associated with residents’ physical activity [77]. Since devising quality measures was not the scope of this study, future studies are required to personalize accessibility measures for different user groups, in order to investigate whether access to the facilities is relevant to various subpopulations. In addition, the use of the negative exponential function to specify our impedance function, instead of using local data on use and travel to sports complexes, has likely affected our results. Also, use of centroids (of postal codes, census blocks, and sports complexes), size of catchments, potential disagreement between measured and self-reported distance are likely to have influenced these results [23,78,79]. As well, because weather and seasonality seem to influence physical activity in outdoor settings [80], it is likely that the date of the survey may have influenced the answers of the respondents. 

## 4. Conclusions

We found that environmental-level factors, such as access to facilities and neighborhood risk from crime, together with individual-level factors such as age, gender, educational attainment, and self-efficacy influence the likelihood that individuals will undertake the recommended levels of physical activity. Consequently, interventions that integrate provision of relevant programs for various population groups (e.g., women, adults of all ages, individuals with higher levels of education with predominantly sedentary occupations, or individuals with lower levels of self-efficacy) with provision of improved recreational facilities may contribute to sport fields becoming catalysts for physical activity. In this respect, sport fields may generate physical activity both on the site and in the neighborhood.
